# Food, Nutrition, Physical Activity and Microbiota: Which Impact on Lung Cancer?

**DOI:** 10.3390/ijerph18052399

**Published:** 2021-03-01

**Authors:** Ersilia Nigro, Fabio Perrotta, Filippo Scialò, Vito D’Agnano, Marta Mallardo, Andrea Bianco, Aurora Daniele

**Affiliations:** 1 Dipartimento di Scienze e Tecnologie Ambientali, Biologiche e Farmaceutiche, Università della Campania “Luigi Vanvitelli”, Via G. Vivaldi 42, 81100 Caserta, Italy; nigro@ceinge.unina.it (E.N.); mallardo@ceinge.unina.it (M.M.); 2 CEINGE-Biotecnologie Avanzate, Via G. Salvatore, 486, 80145 Napoli, Italy; scialo@ceinge.unina.it; 3 Department of Medicine and Health Sciences “V. Tiberio” University of Molise, Via F. De Sanctis, 86100 Campobasso, Italy; dott.fabioperrotta@gmail.com (F.P.); vito.dagnano@studenti.unicampania.it (V.D.); 4 Department of Translational Medical Sciences, Università della Campania “Luigi Vanvitelli”/Hosp. Monaldi, 80131 Naples, Italy

**Keywords:** lung cancer, nutrition, physical activity, microbiota, sarcopenia

## Abstract

Lung cancer still represents the leading cause of cancer-related death, globally. Likewise, malnutrition and inactivity represent a major risk for loss of functional pulmonary capacities influencing overall lung cancer severity. Therefore, the adhesion to an appropriate health lifestyle is crucial in the management of lung cancer patients despite the subtype of cancer. This review aims to summarize the available knowledge about dietary approaches as well as physical activity as the major factors that decrease the risk towards lung cancer, and improve the response to therapies. We discuss the most significant dietary schemes positively associated to body composition and prognosis of lung cancer and the main molecular processes regulated by specific diet schemes, functional foods and physical activity, i.e., inflammation and oxidative stress. Finally, we report evidence demonstrating that dysbiosis of lung and/or gut microbiome, as well as their interconnection (the gut–lung axis), are strictly related to dietary patterns and regular physical activity playing a key role in lung cancer formation and progression, opening to the avenue of modulating the microbiome as coadjuvant therapy. Altogether, the evidence reported in this review highlights the necessity to consider non-pharmacological interventions (nutrition and physical activity) as effective adjunctive strategies in the management of lung cancer.

## 1. Introduction

Lung cancer (LC) remains the leading cause of cancer death worldwide accounting for 14.1 million new cancer cases and 8.2 million deaths per year [1,2]. There are several factors to prevent and manage complications and/or poor prognosis of lung cancer such as nutritional approach and physical activity [3]. Likewise, performance status, body composition, diet and lifestyle are determinant factors influencing overall lung cancer severity and mortality [4]. Interestingly, recent growing evidences demonstrated that sarcopenia, defined as progressive and generalized skeletal muscle disorder, increases likelihood of adverse outcomes, physical disability and mortality among patients with malignancies [5]. Finally, there has been an increasing interest in the role of lung/gut microbiome in lung cancer [6]. Indeed, a growing body of evidence have suggested that a decreased microbiome diversity, specially associated with incorrect life style (nutritional and sedentary regimen) could have an important role in lung cancer formation and progression [7,8].

Treatment options for people with lung cancer vary according to the type of lung cancer, stage of the disease and the patient’s functional status. Treatments can include surgical resection, chemotherapy, radiotherapy, targeted therapy and immunotherapy, which are physically and mentally invasive approaches [9,10,11,12,13]. Besides those treatments, non-surgical/pharmacological approaches are gaining growing attention as adjuvants in the therapy of lung cancer such as dietary plans and physical activity.

Aim of this review is to discuss the complex interaction among the above-mentioned factors in lung cancer patients, dealing with their impact on the prognosis of the disease and response to therapy. In this context, we attempt to analyze the role of nutrition and physical activity on lung cancer establishment and prognosis and the feasibility of nutritional and exercise schemes as strategies to prevent lung cancer. Furthermore, we will discuss how microbiome is associated with lung health and how lung dysbiosis affects tumor progression and disease prognosis.

## 2. General Information about Lung Cancer: Incidence, Risk Factors and Complications

Mostly detected in advanced stages, lung cancer still represents the leading cause of cancer-related death, globally [4] with a mean age at the time of diagnosis of more than 70, lung cancer is considered as an older population disease. Moreover, less than 0.5% of lung cancer related deaths occur in 40 years-old people or younger [5]. Lung tumors are broadly classified into two main categories. Non-small cell lung cancer (NSCLC) represents the vast majority of lung cancer. Histologically, several sub-categories are recognized. The most commonly encountered categories include adenocarcinoma and squamous cell carcinoma, and rarely other non-small cell lung carcinomas (adenosquamous carcinoma, sarcomatoid carcinoma, and others) [14]. Small cell lung cancer (SCLC) represents a neuroendocrine tumor which account for the remaining minority of lung cancer. Whilst the extent of disease is crucial among tumor-related factors, several host-related prognostic factors may determinedly influence the scenario. Firstly, tobacco use, a well-known cause of lung cancer, is crucial for promoting oncogenesis, affecting both disease progression and response to therapy [15]. With a prevalence at time of diagnosis reaching 60%, smoking does represent the number one preventable risk factor for lung cancer genesis [1,2]. Loss of tumor suppressor genes, such as p53, and mutation of oncogenes, such as Kras, may occur as result of chronic exposure to tobacco compounds [3]. Concurrently, smoking habit appears of paramount importance in disease progression and response to therapy [4]. Despite tobacco smoking patients with NSCLC have been reported to experience a better overall response rate (RR) of immunotherapy than no smokers, analysis of KEYNOTE-024 study may indicate survival gains of smoking cessation [5,15]. Moreover, a study on the impact of smoking on NSCLC prognosis has shown that quitting smoking within 3 months of lung cancer diagnosis have increased survival compared to those who continue to smoke [16]. Once again, dysregulated inflammatory response may be crucial [17,18]. In smokers, lower circulating NK cells level may accelerate cancer progression, which in turn can lead to an exacerbation of side effects of cancer treatment [19].

In lung cancer patients, smoking may increase the cellular damage which in turn promotes fatigue and depression. These, in addition to poor nutrition, ageing, sedentariness, finally contribute to the development of sarcopenia. Relationship between smoking and sarcopenia is currently under evaluation. Whilst studies identifies smoking as a potential risk factors for sarcopenia development, this issue needs further elucidation [20,21]. However, the concomitant sarcopenia in lung cancer patients represents an independent risk factor for worse prognosis and increased death risk in lung cancer patients, independently of cancer stage [7].

Lungs are no longer considered sterile and their microbiota are associated with lung wellness. Therefore, a growing interest is concentrated on the lung microbiome that has been linked to lung carcinogenesis and establishment of lung metastasis from other primary cancers [22]. Indeed, preliminary data suggest that both lung and gut microbiota are modulators of the carcinogenic process and seems also to influence the efficacy of immunotherapy affecting the prognosis and survival of the disease. Conversely, profiling of the gut and lung microbiota might reveal dysbiotic signatures associated with delayed tumor outgrowth and favorable responses to immunotherapy [23].

## 3. Muscle Wasting in Lung Cancer Patients: Molecular Mechanisms and Clinical Consequences

In patients with cancer, sarcopenia has been currently regarded as the hallmark of the well-known cancer cachexia, emerging as a highly prevalent phenomenon in several neoplasia. In a meta-analysis, pooled prevalence of sarcopenia in patients with Non-Small Cell Lung Cancer (NSCLC) and Small Cell Lung Cancer (SCLC) has been reported to be 43% and 52%, respectively [24]. Malnutrition, and sarcopenia may both occur in lung cancer [25]. Malnutrition is due to a deficiency of energy intake which can lead to altered body composition and poor clinical outcomes [26]. Cancer sarcopenia is a multifactorial syndrome characterized by the loss of skeletal muscle mass, strength, and/or function determined by several factors (e.g., altered cytokines and systemic inflammation, energy imbalance, adipose tissue depletion) that we will describe later [27].

**Figure 1:**
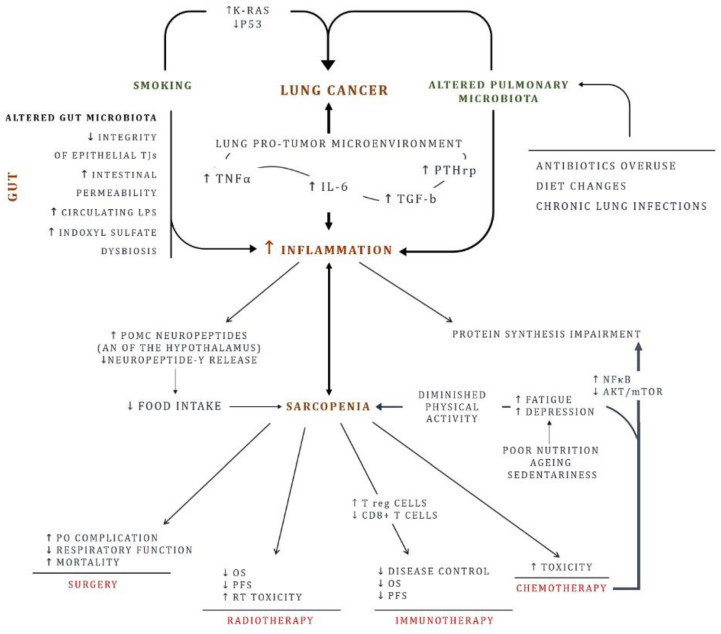
Lung cancer risk factors: focus on inflammation chronic inflammation, and sarcopenia. In addition to smoking, lifestyle (diet change, physical activity, pulmonary and gut microbiota) represents a risk factor postulated to contribute to lung cancer origin. The resulting inflammation ignites the carcinogenesis. On the other hand, inflammation is sustained in the tumor microenvironment, closing the vicious circle. Likewise, gut plays a key role. An impaired intestinal mucosa and an altered gut microbiota promotes inflammation. With time, skeletal muscle weakens. Physical inactivity combined with inadequate diet exacerbates the scenario and sarcopenia development. AKT: Protein kinase B; AN: Arcuate nucleus; IL-6: Interleukin 6; mTOR: mammalian target of rapamycin; NFκB: Nuclear factor kappa-light-chain-enhancer of activated B cells; LPS: Lipopolysaccharides; OS: overall survival; PFS: Progression-free survival; PO: Post-operative; POMC: Pro-opiomelanocortin; PTHrp: Parathyroid hormone-related protein; RT: Radiotherapy; TJs: tight junctions; TGF-β: Transforming growth factor beta; TNFα: Tumor necrosis factor alpha.

Several mechanisms contribute to cancer related sarcopenia. Ubiquitin-proteasome pathway is relevant for degradation of myofibrillar proteins. However, increased lysosomal protease cathepsin-B has been reported in depleted fat-free mass (FFM) lung cancer patients [28,29]. Furthermore, a protein synthesis impairment concurrently occurs [30,31]. In tumor microenvironment, the chaotic interplay between cancer and host cells may lead to an increase of factors and cytokines such as TNFα, IL-6, PTHrp which eventually results in an impaired muscle homeostasis [32]. In Apc^Min/+^ murine models of colorectal cancer, mice with higher levels of IL-6 have been demonstrated to be affected by more severe cachectic syndrome [32]. Similar to IL-6, TNFα appears crucial in cancer-related sarcopenia. Through activation of NF kappa B, it has been reported to hamper protein synthesis and enhance the ubiquitin-proteasome related protein turnover [30,33]. Inflammation status, often trait of cancer, leads to anorexia by activating expression of pro-opiomelanocortin neuropeptides in the arcuate nucleus of the hypothalamus, promoting consequently sarcopenia and cachexia [34,35]. In addition, cytokines reduce neuropeptide Y release, discouraging food intake [24]. In this scenario, chemotherapy may represent a double-edged sword. Despite it remains the cornerstone of advanced lung cancer treatment, evidence is that many chemotherapies may drive sarcopenia via NF kappa B activation and protein kinase B (AKT)/mammalian target of rapamycin (mTOR) downregulation leading to loss of myogenesis [36]. In addition, diminished physical activity secondary to fatigue and impaired food intake contribute to sarcopenia during chemotherapy [35,36,37]. As a result of its global effect, sarcopenia does remarkably impact on lung cancer prognosis [7,38,39]. According to data provided by Yang et al., sarcopenia represents an independent predictor of shorter overall survival (OS) in both stage I-II NSCLC (HR, 3.23; 95% CI, 1.68–6.23) and stage III-IV NSCLC (HR, 2.19; 95% CI, 1.14–4.24) [40]. Nakamura et al. reported a significative difference in major post-operative complications between sarcopenic and non-sarcopenic patients with resected NSCLC. In addition, postoperative major complication has been related with a poor outcome [41]. Interestingly, a higher risk of developing pneumonia observed in sarcopenic patients is thought to be caused by hypercatabolic state and inflammation with increased TNFα, TGF-b, and IL-6, leading to a respiratory function depression and higher mortality [39]. To an uncertain extent, sarcopenia may impact on outcomes of cancer patients treated with radiotherapy [42,43]. Albeit in patients with early-stage lung cancer treated with stereotactic body radiation therapy, increased BMI has been reported to positively impact the OS, the development of sarcopenia, the local failure free survival (LFS) and the distant failure free survival (DFS) [42]. However, in a cohort of 287 patients with definite chemo-radiotherapy (CCRT) treated esophageal cancer, sarcopenia developed post-CCRT has been showed to be associated to shorter OS and PFS [43]. Figure 1 summarizes the above-described processes.

Immune checkpoints inhibitors (ICIs) have dramatically changed the therapeutic landscape of locally advanced and metastatic thoracic malignancies [44,45]. However, it must be noted that prediction of response to agents targeting immune checkpoint inhibitors in elderly population might be modulated from several factors (increased inflammation, reduced emunctory function, gut microbiota) [46,47,48]. Impact of sarcopenia on ICIs response in elderly has currently gained growing awareness. Based on a retrospective study, Nishioka and co-workers showed that patients with advanced NSCLC with sarcopenia were associated with poor outcomes for treatment with immune checkpoints inhibitors (ICIs). CD8+ T cells suppression as well as regulatory T cells (T reg) stimulation in tumor microenvironment has been postulated to induce immunosuppression, hindering immunotherapy response [49]. Similarly, a better progression free survival has been reported in advanced NSCLC patients treated with PD-1 inhibitors without sarcopenia at baseline. In addition, a more favorable overall response has been observed in these patients compared to subject with sarcopenia [50]. In a cohort of NSCLC patients receiving immune-checkpoint inhibitors, Roch et al. found that patients with an evolving sarcopenia showed significantly lower probability of achieving a disease control when compared with controls. Moreover, subject with sarcopenia at the beginning of immunotherapy have been reported to have a numerically shorter median OS when compared with patients without sarcopenia [51].

## 4. Food and Dietary Plans in the Prevention/Control of Lung Cancer

Common phenomena in lung cancer patients are both malnutrition and cancer cachexia [52]. The prevalence of malnutrition in lung cancer patients ranges from 34.5 to 69%, with the highest incidence in more severe patients and in those undergoing chemotherapies, immunotherapy and/or radiotherapy [53]. On the other hand, inactivity represents a major risk for loss of functional pulmonary capacities in lung cancer patients [3]. Nutritional counselling, planning of meals and use of supplements are essential approaches to counteract malnutrition and sarcopenia in lung cancer. In fact, a nutritional and life-style counselling approach is recommended to control chemotherapy response, sarcopenia, prognosis and survival of the lung cancer patients. Tanaka et al. (2018) demonstrated that an early nutritional intervention with a dietary counselling in lung cancer patients receiving chemotherapy efficiently counteracts weight loss and sarcopenia [54]. However, many patients do not achieve recommended dietary intake even after nutritional counselling [55]. The main nutritional approaches to prevent and treat cancer sarcopenia are: an adequate energy intake; an adequate supply of protein for maintenance or gain of muscle; use of supplements.

An adequate protein intake can reduce the incidence and severity of sarcopenia in cancer patients [56]. It has been demonstrated that a dietary program with energy and protein rich meals and snacks can improve muscle strength and performance status of lung cancer patients [57,58].

The use of supplements in the diet for cancer patients experiencing muscle loss is becoming a very popular approach. Several products might be useful in contrasting sarcopenia during cancer (Branched-chain amino acids, carnitine, fish oil, Eicosapentaenoic acid (EPA), vitamins and mineral, [59]. Specifically, in lung cancer, supplementation of diet with EPA and PUFA improves the maintenance of weight and muscle mass in advanced NSCLC patients undergoing chemotherapy as well as physical and cognitive functioning [60,61,62].

Increasing attention has been focused on the possible use of oral ghrelin receptor (G-protein coupled receptor, GHSR-1a) agonists such as anamorelin and HM01 with the aim of exploiting the ghrelin’s orexigenic capacity [63]. Anamorelin, a ghrelin receptor agonist, has been demonstrated to be able to significantly increase lean body mass [64]. Two completed clinical trials (ROMANA1 and 2, NCT01387269 and NCT01387282, respectively), performed on lung patients with inoperable stage III or IV non-small-cell lung cancer and cachexia, demonstrated that anamorelin induces an increase in lean body mass, without modification in the handgrip [65]. A third trial from the same authors, ROMANA3 (NCT01395914) has been completed confirming the improvements in body weight and anorexia-cachexia symptoms observed in the original trials, and demonstrating a well toleration to anamorelin administration [66]. There are currently two ongoing clinical trials (NCT03743064 and 03743051) investigating the use of anamorelin to treat non-small cell lung cancer-associated weight loss. Both trials report changes in weight although a definitive result has not been reached. On the contrary, in vitro and vivo data are available about HM01 effects on cachexia but no clinical trials are available yet [67,68].

Regarding the molecular mechanisms underlying anamorelin effects, Garcia and colleagues found the it significantly increases GH, IGF-1 and IGFBP-3 levels with consequent body weight gain [69,70]. A very recent study compared the two ghrelin receptor agonists anamorelin (non-BBB penetrant) and HM01 (BBB penetrant), demonstrating that HM01 enhances hypothalamic neuronal activation and increases cumulative food intake compared to ghrelin and anamorelin [71]. The authors also demonstrated that HM01 and anamorelin exert potent effects on calcium mobilization, however anamorelin is potentially more susceptible to treatment-induced tolerance than HM01 due to recruitment of β-arrestin and GHSR-1a internalization [71].

## 5. Effects of Food and Dietary Plans on Lung Cancer

As said above, body composition and eventually the presence of sarcopenia are crucial factors determining the risk, response to therapy and therefore the prognosis of lung cancer patients. Considering nutritional status as a determining factor of the body composition, in recent years growing attention has been paid to the choice of dietary plans as well as to performing physical activity. Dietary schemes as well as specific foods-enriched diet influence the predisposition towards cancer disease and the response to therapies and therefore the prognosis. The main molecular processes regulated by specific diet patterns, functional foods and physical activity in relation to cancer are the inflammation and oxidative stress. In the next paragraphs, we report the main dietary schemes associated to body composition, response to therapy and prognosis of lung cancer patients: caloric restriction, PUFA-enriched diets, Dietary Approaches to Stop Hypertension (DASH), fibers-enriched diet and diary-enriched diet. Since a considerable variety of bioactive ingredients have been identified in foods, we will also report interesting data for single compounds.

### 5.1. Caloric Restriction

It is widely believed that calorie restriction can extend the lifespan of model organisms and protect against aging-related diseases, such as lung cancer. In breast cancer, Simone et al. demonstrated that caloric restriction can augment the effects of radiation therapy as well as chemotherapy in a mouse model of breast cancer [72]. Interestingly, Safdie et al. analyzed patients diagnosed with a variety of malignancies (one with lung cancer) that voluntarily fasted prior to (48–140 h) and/or following (5–56 h) chemotherapy reporting a reduction in fatigue, weakness and gastrointestinal side effects while fasting [73]. The molecular mechanism of caloric restriction action is mainly related to the decrease of chemotherapy-induced inflammation and induction of energy stress resulting in increased efficacy of therapy. In lung cancer, Caiola et al. suggested, through in vitro studies, that caloric restriction regimens may sensitize NSCLC lesions carrying KRAS mutation and LKB1 loss to cytotoxic chemotherapy through induction of energy stress [74]. Resveratrol has been proposed as an active molecule mimicking the effects of caloric restriction which may have beneficial effects against numerous diseases such as type 2 diabetes, cardiovascular diseases, and cancer [75]. The positive effects in cancer are related to by the inhibition of oxidative stress, inflammation, aging, and fibrosis [76,77]. In lung cancer, and more widely, in lung diseases resveratrol represents a promising natural compound to be used in association with other drugs [78]. Although it is clear that resveratrol has shown excellent anti-cancer properties, most of the studies were performed in vitro or in pre-clinical models. Few clinical trials have been developed on the administration of resveratrol in cancer patients [79,80]. In addition, resveratrol in its current form is not ideal as therapy because, even at very high doses, it has modest efficacy and many downstream effects [81]. The identification of the cellular targets responsible for resveratrol effects would help in the development of target specific therapies based on this drug.

### 5.2. PUFA-Enriched Diets

Inflammation plays a central role in cancer etiology and can be modulated by diet. Indeed, diet and inflammation have been suggested to be important risk factors for several cancers including lung cancer. Shivappa et al. examined the ability of the dietary inflammatory index (DII^®^) to predict lung cancer [82]. The authors define DII a diet quality index based on the literature linking foods and nutrients with inflammatory biomarkers. The DII is non-significantly associated with risk of lung cancer in non-smoker patients but a strong association is present for subjects with a history of smoking [82]. Animal studies have shown that polyunsaturated fatty acids (PUFAs) have antineoplastic and anti-inflammatory properties [83]. Two population-based cohort studies, the Shanghai Women’s Health Study (SWHS) and Shanghai Men’s Health Study (SMHS) with a total of 121,970 study participants investigated that the association of specific types of dietary PUFA intakes and lung cancer risk [84,85]. Total, saturated and monounsaturated fatty acid intakes are not significantly associated with lung cancer risk. However, interestingly, PUFAs intake and the ratio of n-6 PUFAs to n-3 PUFAs are inversely associated with lung cancer risk, particularly among never-smokers. This observation highlights an important public health impact of PUFA intakes in lung cancer patients. The molecular mechanism at the basis of such effects of PUFAs appear to be anti-inflammatory and anti-oxidative, both able to improve the nutritional status of cancer patients [86,87,88]. In patients with a diagnosis of advanced inoperable NSCLS and undergoing chemotherapy, PUFAs consumption increases body weight, reduces C-reactive protein and IL-6 levels during chemotherapy, evidencing a clear anti-inflammatory action of PUFAs [83]. Concerning oxidative status, PUFAs avoid plasma reactive oxygen species levels increase during chemotherapy [83].

Starting from these observations, the use of supplements in the diet might also be considered for lung cancer patients. Sánchez-Lara et al. compared the effect of an oral EPA enriched supplement with an isocaloric diet on nutritional, clinical and inflammatory parameters in advanced NSCLC patients receiving paclitaxel and cisplatin/carboplatin treatment [60]. Compared with baseline, patients receiving the EPA supplement gained lean body mass compared with a loss of in the control. In addition, patients with NSCLC receiving ONS-EPA significantly improves energy and protein intake, body composition. and decreased fatigue, loss of appetite and neuropathy [60].

Overall, these data demonstrate that the continual assumption of PUFAs determines an anti-inflammatory and anti-oxidative action that could be considered a preliminary goal in anti-cachectic therapy.

### 5.3. DASH

DASH is an eating plan to lower or control high blood pressure. The DASH diet emphasizes the consumption of foods that are lower in sodium as well as foods rich in potassium, magnesium and calcium—nutrients that help lower blood pressure.

Several investigators have proposed a protective association between DASH style diet and a reduced risk as well as a reduced mortality from many cancer types [89]. Regarding lung cancer, it has been reported that high adherence to DASH is associated with a decreased mortality. Anic et al. analyzed four diet quality indices—Healthy Eating Index–2010 (HEI-2010), Alternate Healthy Eating Index–2010 (AHEI-2010), alternate Mediterranean Diet score (aMED) and Dietary Approaches to Stop Hypertension (DASH)—and lung cancer risk [90]. The authors observed that a higher diet quality, as measured by the scores, is associated with a significant lower risk of lung cancer, in particular among former smokers where the statistical power was greater than in non-smokers [90]. In addition, when stratifying by histology type, they found an inverse association with adenocarcinomas and squamous cell carcinomas for all diet indices, but not with small cell carcinomas. Although smoking is the factor most strongly associated with lung cancer, growing body of evidence suggest that diet may have a modest role in reducing lung cancer risk.

### 5.4. Fibers-Enriched Diet

Fruit, vegetables and certain components of plant foods, such as fiber, are associated with a reduction in systemic inflammation, obesity and metabolic syndrome, even after adjustment for important confounding variables [91]. In addition, high fiber intake has long been thought to protect against several types of cancer [92]. The mechanisms for those various health benefits seem to be linked to the modulation of the gut microbiota and metabolic pathways that fibers can induce [93]. Fiber intake is inversely associated with lung cancer risk after adjustment for status and pack-years of smoking and other lung cancer risk factors in 1,445,850 adults from studies that were conducted in the United States, Europe, and Asia [94]. Similarly, Miller et al. studied data from 478,021 individuals included in the EPIC study, and recruited from 10 European countries and who completed a dietary questionnaire [95]. After adjustment for age, smoking, height, weight and gender, there was a significant inverse association between fruit consumption and lung cancer risk in lung cancer patients. The association was strongest among current smokers at baseline [95].

Considering subtypes of lung cancer, Büchner et al., 2010 observed an inverse association between the consumption of fibers and risk of lung cancer without a clear effect on specific histological subtypes of lung cancer [96].

On the other hand, considering different sources of fibers, Bradbury et al., 2014 reported that the risk of cancer of the lung was inversely associated with fruit intake but was not associated with vegetable intake [97]; however, this association with fruit intake is restricted to smokers. In accordance with this data, Büchner et al. analyzed the effects of fruits and vegetables during a follow-up of 1830 incident cases of lung cancer; a 100 g/day increase in fruit and vegetables consumption was associated with a reduced lung cancer risk [96]. In addition, different sources of fibers do not alter positive effects, as demonstrated by Baldrick et al. that found beneficial effects in ex/smokers following a diet with high intake of fibers from legumes through anti-inflammatory mechanisms [98].

An association has been also found between total fiber intake and decreased COPD risk suggesting a beneficial impact on general lung health [99,100].

### 5.5. Diary-Enriched Diet

Dairy foods (DFs) contain complex ingredients that could affect different diseases [101]. Milk fat is a natural product containing essential nutrients as well as fatty acids and other food factors with reported anti-cancer potential [102]. The effects of dairy products on human health have been studied for years. In adults, intake of dairy products was shown to improve body composition and facilitate weight loss during energy restriction [103]. However, the relationship between dairy products as well as calcium intake and the risk of lung cancer is still inconclusive. Kubik et al., 2004, in a case-control study, investigated the relationship between diet and the risk of lung cancer among non-smokers as well as smokers’ women finding protective effects of a frequent intake of milk/dairy products only among smoking group [104]. On the contrary, Mettlin et al., 1989 reported that subjects consummating whole milk three or more times daily had a two-fold increase in lung cancer risk compared to those who reported never drinking whole milk [105]. Yang et al., 2016 analyzed 32 studies and, after stratifying by potential confounders, found that the intake of dairy products or calcium was not statistically associated with the risk of lung cancer [106]. Similarly, Thorning et al., 2016 analyzed milk and dairy intake among cancers describing an inverse association with colorectal cancer, bladder cancer, gastric cancer and breast cancer, and not associated with risk of pancreatic cancer, ovarian cancer or lung cancer [103]. Regarding calcium consumption, a possible role for increasing dietary calcium intake in lung cancer prevention has also been suggested among non-smokers subjects, especially in populations with relatively low calcium intake [107].

When considering individuals with lactose intolerance, characterized by low consumption of milk and other dairy product, J Ji et al., 2015 found decreased risks of lung, breast and ovarian cancers, but the decreased risks are not found in their family members, suggesting that the protective effects against these cancers may be related to their specific dietary pattern [108].

In a mix dietary pattern, characterized by higher frequency of dairy, fruit, vegetables, whole meal bread, fish and juices consumption, Krusińska et al. found an association between this dietary pattern and breast or lung cancer prevalence, irrespective of age, socioeconomic status, physical activity, smoking, alcohol abuse and type of cancer in Polish adults from north-eastern Poland [109].

## 6. Impact of Physical Activity in the Prevention and Management of Lung Cancer

Recently, growing evidence supports activity’s benefit in chronically ill patients [110]. Indeed, in chronic lung diseases such as COPD and Cystic Fibrosis, physical activity has proven consistent beneficial effects in terms of respiratory function (FEV1%, FVC, decreased dyspnea and fatigue, improvement in shortness of breath) as well as in terms of quality of life (cognitive functions) [111,112,113,114].

Respect to lung cancer, physical activity has been described as a preventive factor able to reduce the risk as well as a non-pharmacological approach to manage the disease ameliorating the carcinogenesis risk, the chemotherapy response and finally prognosis and survival [115,116,117,118]. Indeed, home-based exercise is a beneficial approach to improve symptoms and quality of life of patients with lung cancer [119]. On the other hand, the risk of an adverse event with exercise is low, reinforcing the necessity for lung cancer patients to perform physical activity and keep active [120,121]. Increased physical activity and resistance exercise is a cornerstone of the management of sarcopenia [122] while physical inactivity represents a major risk for loss of functional capacities. Exercise and physical activity can reduce inflammation [123] as well as can induce molecular signaling pathways that support building muscle mass, and stimulate beneficial metabolic adaptations [124]. In lung cancer, physical activity and exercise are non-pharmacological interventions that have been shown to improve fatigue, quality of life, pulmonary function, muscle mass and strength and psychological status [3]. Previous interventional studies that included strength assessment as a result of resistance training in patients with lung cancer reported positive effects of physical activity [125,126,127,128,129,130,131]. Conversely, only few studies reported no effects of physical activity on muscle strength in lung cancer patients [132,133,134]. Salhi et al. investigated the impact of the physical activity on muscle mass in lung cancer patients performing a 12-week rehabilitation program compared to sedentary patients demonstrating that active lung cancer patients preserved muscle mass while sedentary patients experienced muscle loss [135].

Summarizing, the biological effects of exercise in the context of lung cancer patients are: improvements of fatigue, enhancement of pulmonary functions, maintenance of muscle strength, amelioration of sleep quality, altogether resulting in an improved quality of life [3]. Several studies reported the effects of exercising in lung cancer patients according to the possibility of performing surgery [136]. In patients with operable lung cancer, preoperative exercise decreases the risk of post-operative pulmonary complications and improves the post-operative rehabilitation [132,137]. Preoperative high-intensity training in frail old patients undergoing pulmonary resection for NSCLC symptoms with a better rehabilitation again [137].

In previous studies, the efficacy of preoperative rehabilitation in patients undergoing lung resection for NSCLC was proved in both in-hospital and home-based settings [136,138]. Indeed, performing exercise training in the post-operative phases improves muscle strength and respiratory symptoms with a better rehabilitation again [139]. In patients with inoperable lung cancer, exercise training helps in maintaining lung functions and muscle strength, reducing the risk of sarcopenia [140].

The molecular mechanisms at the basis of exercise effects in lung cancer are multiple and complex and not still not fully understood. An immunomodulatory effect of exercise has been previously reported as the main molecular mechanism regulated by exercise in several conditions as well as in lung cancer [3,141]. Indeed, exercise can increase the levels of proinflammatory cytokines in cancer microenvironment through the up-regulation of natural killer cells, lymphocytes and dendritic cells, thus resulting in suppression of cancer growth [142]. These mechanisms at least in part explain the anti-cancer effects of exercise. A further important mechanism of action of the beneficial effects of physical activity is the regulation of angiogenesis [143,144].

There is the need of clinical trials investigating multimodal interventions including exercise and nutrition to target sarcopenia in lung cancer patients. More importantly, it seems to be of fundamental importance the evaluation of individual needs to efficiently counteract the progressive weight loss in sarcopenic patients.

## 7. Microbiota and Lung Cancer

The development of next-generation sequencing (NGS) has completely changed the idea of lungs as organs in a condition of sterility, proving that also healthy lungs are colonized by different bacterial communities [145] important in shaping the immune system [146] and developing tolerance to allergens [147]. Moreover, many studies seem to confirm an association between alteration in lung microbiome composition (dysbiosis) and lung cancer although we are still far from elucidating the molecular links. For instance, by using Bronchoalveolar lavage fluid (BALF) from lung cancer patients many studies have demonstrated an abundance of diverse species of bacteria such as TM7-3, Capnocytophaga and Sediminibacterium while a decrease in others such as Microbacterium and Stenotrophomonas compared to control groups [148,149,150]. At the same time, gut dysbiosis has been associated with different lung pathologies, such as asthma [151], chronic obstructive pulmonary disease (COPD) [152] and lung cancer [153]. Furthermore, antibiotic treatment in lung cancer patients started before chemotherapy or immunotherapy has been associated with less efficacy and reduced survival compared with subject that did not received antibiotics [154]. Routy et al. demonstrated that fecal microbiota transplant (FMT) from lung cancer patients that responded to PD-1/PD-L1 immunotherapy could restore the response to the immunotherapy in antibiotic treated or germ free mice, identifying the bacterium Akkermansia muciniphila and Alistipes indistinctus as responsible for this effect [155]. Interestingly, Tsay and col. have recently demonstrated that lower airways dysbiosis in patients with NSCLC stage IIIB-IV are more enriched with oral microbiota and this correspond to an increased cancer progression and worst outcome. Furthermore, they show both in patients and in a preclinical model, that Veillonella parvula was strongly associate with an increased inflammatory phenotype driven by IL-17 and an upregulation of ERK, MAP and PI3K pathways [156].

The control of clinical prognosis and response to immune therapy by lung and gut microbiome seems to be strictly related to induction of inflammatory processes as well as to inhibition of immune checkpoints; up-regulation of several cytokines has been demonstrated in dysbiosis mice while specific microbiome signatures are associated with anti-tumor activity and PD-1 blockade response [157].

Altogether this evidence highlights that dysbiosis of lung and/or gut microbiome, as well as their interconnection (the gut-lung axis), play a key role in lung cancer formation and progression, opening to the interesting avenue of modulating the microbiome as coadjuvant therapy. Dietary patterns and regular physical activity may represent non-pharmacological approaches modulating microbiome health and therefore the risk of lung cancer [22]. Lifestyle, nutrition, and geographical provenance are all factors that possibly interfere with the above-mentioned mechanisms and can contribute to shape the lung and gut microbiome [158]. Particularly, it is well known that people following different diet regimes have different gut microbiome composition [158], and there is a correlation between high consumption of meat and fat with the risk to develop lung cancer [159,160,161]. For instance, it has been seen that specific metabolites such as omega-6 (ω6) polyunsaturated fatty acid (PF) and ω3PF can have opposite effect on cancer progression [162]. In fact, while ω6PF have been shown to induce pro-inflammatory phenotype increasing cancer progression, ω3PF have an anti-proliferative and apoptotic effect on different lung cancer cells [163]. This effect was shown to be dependent on ROS generation and autophagy induction since treatment with N-acetyl cysteine inhibited this phenotype [164,165]. Furthermore, the consumption of a high fiber diet and yogurt, rich in prebiotic and probiotic respectively have been associated with a reduced risk of lung cancer [94]. Indeed, it has been demonstrated that short-chain fatty acid released in the circulation by the effect of intestinal microbiota plays a key role in the regulation of the immune response contributing to the homeostasis maintenance of different organ such as the lung [166]. However, the association between microbial dysbiosis and lung cancer is not clearly understood, future studies involving larger cohorts and metagenomics, or metabolomics, may elucidate the correlations between gut microbiota and lung cancer development.

Physical activity is a modifying factor preventing different pathologies such as respiratory, cardiovascular, neuroendocrine and muscular diseases as well as cancer. Yet, we still have little information of the beneficial effects of physical exercise on gut microbiome health but it seems that exercise-derived benefits on microbiome diversity can beneficially influence other tissues and body organs. Endurance exercise may modulate GIT immune-inflammatory and redox responses, GIT permeability, motility and consistency with positive effects towards inflammation and oxidative stress, both processes related to carcinogenesis [167,168,169]. Evidence for a protective role of exercise through microbiome modulation has been described in in colon cancer. Woods et al. demonstrated that acute and chronic exercise invokes changes in the microbiome and metabolome that may be beneficial to the prevention or treatment of IBD and colon cancer [170]. Regarding lung cancer, there are demonstrations that the gut microbiome of lung cancer patients is altered significantly compared with healthy individuals [171] but further studies are needed to fully understand whether physical activity can affect prevention or treatment of lung cancer through modulation of gut microbiome.

Intriguingly, skeletal muscle may represent the missing link between gut and cancer outcomes. It has been indeed demonstrated not only the magnitude of the impact of gut ecology in inflammation genesis and skeletal muscle homeostasis (gut-muscle axis); it has been also confirmed the influence of skeletal muscle on cancer prognosis. An impaired integrity of epithelial thigh junctions and the increased intestinal permeability promote the passage of microbial products, such as endotoxin (LPS) and the tryptophan derivative indoxyl sulfate (IS), into the circulation, leading to the inflammation cascade to occur [172]. In addition, both IS and LPS induce inflammatory cytokine expression (IL-6 and TNF-α) which in turn may sustain sarcopenia [173]. Moreover, IS has been reported to induce the expression of muscle atrophy markers, such as myostatin and atrogin-1. [174] Lastly, dysbiosis may play a key role in the development of both inflammation and sarcopenia. Aged and compromised gut ecosystem may prompt to an excessive inflammatory status and to a defective attempt to counteract adverse microbes [175,176]. Conversely, Bindels et al. reported a reduced expression of muscle atrophy markers (Atrogin-1, MuRF1, LC3 and Cathepsin L) in the gastrocnemius and in the tibialis following oral restoration of *Lactobacillus reuteri* and *L. gasser*. Concurrently, a decrease of inflammatory cytokines (IL-6, monocyte chemoattractant protein-1, IL-4 and granulocyte colony-stimulating factor) has been reported [177]. Interfering with these mechanisms may contribute to reduce the risk of sarcopenia related to alterations of gut microbiome in lung cancer patients.

## 8. Conclusions

**Figure 2:**
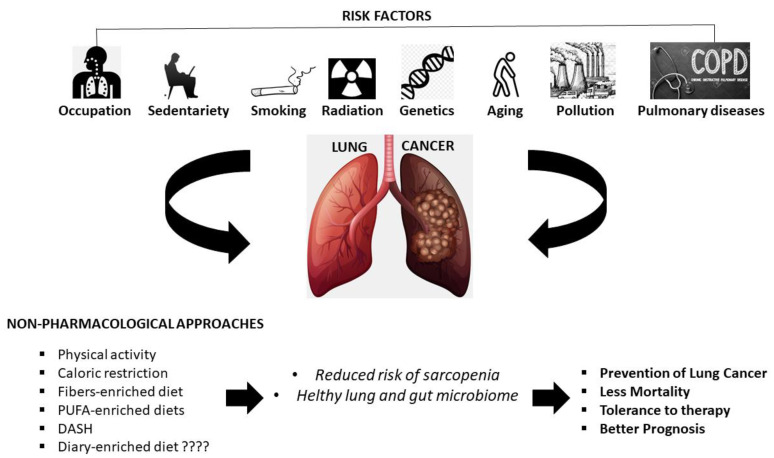
Non-pharmacological approaches acting on lung cancer incidence and management. Regular physical activity, caloric restriction, fibers-enriched diet, PUFA-enriched diets, DASH and diary-enriched diet may reduce the risk of sarcopenia and influence lung and gut microbiome resulting in decreased cancer incidence, reduced mortality, tolerance to therapy and a better prognosis.

The consumption of a healthy, anti-inflammatory diet together with regular physical activity is fundamental in reducing the risk of lung cancer, especially in current and former smokers. Table 1 summarizes the main results obtained from human studies. Physical activity and dietary plans can also be used as an adjunctive therapy to improve the management and reduce poor prognosis of lung cancer, especially for patients undergone sarcopenia. Furthermore, lung and gut microbiome health, greatly influenced by food and physical activity, have a great impact on cancer prevention, and on the response to therapy and prognosis. Vice versa, microbiome dysbiosis can promote cancer progression through different pathways such as increasing inflammation, dysregulating the immune response and alteration in metabolism. Collectively, the data presented in this review provided insight into the necessity to introduce non-pharmacological interventions (nutritional plans + physical activity) in the therapeutic schemes of lung cancer patients in combination with conventional therapies (Figure 2). Performing a regular physical activity and reducing sedentary behaviors together with a reasoned selection of foods and dietary plans is likely able to influence the prognosis of the disease. Positive effects of those approaches likely pass through the influence of biological processes crucial in the carcinogenesis such as inflammation, immunity and lung- and gut-microbiome modulation. Future studies in large cohorts of patients with a variety of disease stage and individual backgrounds will help in developing and modeling the proposed innovative management intervention in a person-centered way.

## Figures and Tables

**Table 1:** Main results from human studies summarizing the effects of dietary and physical activity interventions in lung cancer patients.

Authors	Type of Intervention	Main Results	Enrolled Subject
Leedo et al., 2017 [57]	Protein-rich meals	Increased protein intakes are associated with improved quality of life, functional score, hand grip strength.	40 malnourished lung cancer patients
Sanchez-Lara et al., 2014 [60]	Supplementation with oral eicosapentaenoic acid (EPA) enriched supplement	Lean body mass gain, decrease of fatigue, increase in appetite, decrease of neuropathy, no difference in overall survival	92 patients with advanced non-small cell lung cancer (NSCLC)
Murphy et al., 2011 [61]	Fish oil supplementation	Gain or maintenance of muscle mass, no difference in total adipose tissue	40 non-small cell lung cancer patients
van der Meij et al., 2012 [62]	Protein- and energy-dense oral nutritional supplement containing n-3 polyunsaturated fatty acids	Improved quality of life parameters, physical and cognitive function, no difference in handgrip strength, higher physical activity level	40 patients with stage III NSCLC
Temel et al., 2016 [64] Currow et al., 2018 [65]	Anamorelin 100 mg orally once daily for 12 weeks	Improvements in body weight and anorexia-cachexia symptoms. no difference in handgrip strength	979 and 513 patients with inoperable stage III or IV non-small-cell lung cancer and cachexia
Safdie et al., 2009 [73]	Fasting prior to (48–140 h) and/or following (5–56 h) chemotherapy	Reduction in fatigue, weakness, and gastrointestinal side effects while fasting	10 cases with a variety of malignancies (1 lung cancer)
Shivappa et al. [83]	Examination of the ability of the dietary inflammatory index (DII) to predict lung cancer	A pro-inflammatory diet, as shown by higher DII scores, is associated with an elevated risk of lung cancer for subjects with a history of smoking.	1851 lung cancer cases
Luu et al., 2018 [84]	High intake of specific types of dietary Polyunsaturated fatty acids (PUFA) intakes	Total, saturated and monounsaturated fatty acid intakes were not significantly associated with lung cancer risk. Total PUFAs and the ratio between n-6 PUFAs and n-3 PUFAs were inversely associated with lung cancer risk. DHA intake was positively associated with lung cancer risk.	121,970 study participants
Anic det al., 2019 [90]	Evaluation of four diet quality indices: Healthy Eating Index-2010, Alternate Healthy Eating Index-2010, alternate Mediterranean Diet score and Dietary Approaches to Stop Hypertension	A higher diet quality, as measured by the scores, is associated with a significant lower risk of lung cancer, in particular among former smokers	460,770 participants
Yang et al., 2020 [94]	High dietary fiber and yogurt consumption	Both fiber and yogurt intakes were inversely associated with lung cancer risk after adjustment for status and pack-years of smoking and other lung cancer risk factors: hazard ratio, 0.83 (95% CI, 0.76–0.91) for the highest vs. lowest quint	1,445,850 adults
Miller et al., 2004 [95]	Personal interviews regarding fruit and vegetable intake	After adjustment for age, smoking, height, weight and gender, there was a significant inverse association between fruit consumption and lung cancer risk	478,021 individuals
Büchner et al., 2010 [96]	Evaluation of diet diversity scores (DDS) used to quantify the variety in fruit and vegetable consumption.	With increasing variety in vegetable subgroups, risk of lung cancer decreases. This inverse association is restricted to current smokers	1613 lung cancer patients
Bradbury et al., 2014 [97]	Personal interviews regarding fruit, vegetable, or fiber consumption	The risk of cancer of the lung was inversely associated with fruit intake but was not associated with vegetable intake; this association with fruit intake was restricted to smokers and might be influenced by residual confounding due to smoking.	>500,000 participants
Kubick et al., 2004 [104]	Personal interviews	Among smoking women, protective effects were observed for frequent intake of milk/dairy products	435 lung cancer cases
Mettlin 1989 [105]	Evaluation of consumption of milk, coffee, tea, soft drinks and alcoholic beverages	Subjects reporting consumption of whole milk three or more times daily had a two-fold increase in lung cancer risk compared to those who reported never drinking whole milk	569 lung cancer patients
Yang et al., 2016 [106]	Dairy products as well as calcium intake	the intake of dairy products or calcium was not statistically associated with the risk of lung cancer	Analysis of 12 cohort studies and 20 case-control studies
Takata et al., 2013 [107]	Intakes of calcium and related minerals, assessed through a food frequency questionnaire,	Intakes of calcium, phosphorus, and the calcium-to-magnesium (Ca:Mg) ratio were inversely associated with lung cancer risk	71,267 female nonsmokers
Ji et al., 2015 [108]	Avoid milk or dairy products in individuals with lactose intolerance	People with lactose intolerance, characterized by low consumption of milk and other dairy products, had decreased risks of lung cancer	22,788 individuals with lactose intolerance
Krusińska et al., 2017 [109]	Analysis of food consumption frequency for 21 selected foods using the Questionnaire of Eating Behaviors (QEB)	A strong inverse relation between a ‘Prudent’ dietary pattern, characterized by higher frequency of dairy, fruit, vegetables, wholemeal bread, fish and lung cancer prevalence	80 men with lung cancer
Yang et al., 2020 [116]	Home-based exercise	Home-based exercise significantly improved exercise capacity, reduced cancer-related fatigue, insomnia, anxiety, and depression, and improved quality of life. However, it did not significantly reduce pain, appetite loss, and coughing symptoms	Review of 14 published trials, involving 694 patients in total
Tardon et al., 2005 [118]	Moderate leisure-time physical activity (LPA)	Higher levels of LPA protect against lung cancer.	meta-analysis of all relevant reports published from 1966 through October 2003
Hoffman et al., 2014 [119]	Brief Fatigue Inventory (BFI) measuring CRF severity, and the M.D. Anderson Symptom Inventory	Participants’ CRF severity scores were reduced to mild levels while the mean number of symptoms decreased from 10.4 post-surgery to 7.0 at week 6 with lower levels of severity and interference.	Seven post-thoracotomy NSCLC patients
Granger et al., 2013 [121]	Exercise training	Intervention was safe and associated with positive trends of improvement in some health-related quality of life (HRQoL) domains.	Fifteen lung cancer patients
Kuehr et al., 2014 [125]	8 weeks exercise at least five times per week	Exercise training is feasible in advanced and metastatic NSCLC patients during anticancer treatment. Endurance and strength capacity improved over time, indicating the rehabilitative importance	40 patients with predominantly advanced NSCLC receiving simultaneous or sequential radiochemotherapy or chemotherapy
Quist et al., 2015 [126]	6-week hospital-based supervised, structured, and group-based exercise program	The exercise program improved physical capacity (VO2peak), functional capacity, anxiety level, and emotional well-being	114 patients with advanced stage lung cancer
Arbane et al., 2011 [127]	Physical activity intervention (twice daily training plus usual care)	Training after thoracotomy successfully prevented the fall in quadriceps strength	53 (28 male) patients attending thoracotomy for lung cancer
Edvardsen et al., 2015 [128]	High-intensity endurance and strength training (60 min, three times a week, 20 weeks), starting 5–7 weeks after surgery	High-intensity endurance and strength training is well tolerated and induces clinically significant improvements in peak oxygen uptake, muscular strength, total muscle mass, functional fitness and HRQoL.	61 randomized lung cancer patients
Henke et al., 2014 [129]	Conventional physiotherapy or special physiotherapeutic training	Significant differences were detectable in the Barthel Inde, in physical functioning, pain in arms or shoulder, peripheral neuropathy, cognitive functioning, in the 6-min walking test, stair walking, strength capacity, and in the patient’s dyspnea perception during submaximal walking activities	46 lung cancer patients
Sommer et al., 2016 [130]	The preoperative intervention consisted of a home-based exercise program, while the postoperative exercise program comprised a supervised group exercise program involving resistance and high-intensity interval cardiorespiratory exercise 2 h weekly for 12 weeks	No adverse events were observed.	40 patients with biopsy-proven NSCLC stages I to IIIa referred for surgical resection
Quist et al., 2012 [131]	Supervised, hospital-based muscle and cardiovascular group training and individual home-based training.	Improvements in estimated VO(2peak) and six-minute walk distance (6 MWD) as well as increased muscle strength measurements. Significant improvements in the “emotional well-being” parameter (FACT-L) while there were no significant changes in HRQOL.	25 patients with non-small cell cancer (NSCLC) stage III-IV and four patients with extensive disease small cell lung cancer (SCLC-ED)
Cavalheri et al., 2017 [132]	8 weeks of supervised exercise training (exercise group) or 8 weeks of usual care (control group).	Compared with any change seen in the control group, those in the exercise group demonstrated greater gains in the peak rate of oxygen consumption.	17 lung cancer participants
Arbane et al., 2014 [133]	Usual care or a hospital plus home exercise program.	A hospital plus home exercise program showed little benefit in unselected patients with NSCLC following surgery.	131 subjects with NSCLC admitted for curative surgery
Codima et al., 2021 [134]	Exercise protocols consisting of different combinations of strength, aerobic, and inspiratory muscle training.	Exercise can lead to improvements of symptoms and of quality of life in lung cancer survivors. Providing resistance training combined with high-intensity interval aerobic exercise after lung resection seems to be particularly effective.	10 published studies (835 participants)
Salhi et al., 2014 [135]	12-week rehabilitation training program	Muscle mass and strength: (1) are decreased at presentation in a substantial part of lung cancer patients; (2) are significantly negatively affected by radical treatment and (3) completely recover after a 12-week structured rehabilitation program,	45 lung cancer patients
Perrotta et al., 2019 [136]	Three-week high-intensity pulmonary rehabilitation programs	Significant improvements in the mean peak oxygen uptake	25 consecutive patients with chronic obstructive pulmonary disease (COPD) prior to undergoing lung surgery for NSCLC
Rispoli et al., 2020 [138]	2–4-week pulmonary rehabilitation programs	Preoperative pulmonary rehabilitation significantly enhances clinical status of COPD patients before NSCLC resection.	83 COPD patients with NSCLC
Brocki et al., 2015 [139]	2 weeks of inspiratory muscle training (IMT)	Two weeks of additional postoperative, compared with standard physiotherapy alone, did not preserve respiratory muscle strength but improved oxygenation in high-risk patients after lung cancer surgery.	34 lung cancer patients
